# Association between Common Genetic Variants and Polycystic Ovary Syndrome Risk in a Chinese Han Population

**DOI:** 10.4274/jcrpe.2784

**Published:** 2016-12-01

**Authors:** Ying Sun, Yi Yuan, Hua Yang, Jingjie Li, Tian Feng, Yongri Ouyang, Tianbo Jin, Ming Liu

**Affiliations:** 1 Xi’an Jiaotong University School of Medicine, Department of Pathology, Xi’an, China; 2 Han Zhong Central Hospital, Clinic of Obstetrics and Gynecology, Han Zhong, China; 3 Northwest University School of Life Sciences, Xi’an, China; 4 Xi’an Jiatong University Second Affiliated Hospital, Department of Obstetrics and Gynecology, Xi’an, China

**Keywords:** Polycystic ovary syndrome, single nucleotide polymorphism, Chinese Han women

## Abstract

**Objective:**

Polycystic ovary syndrome (PCOS) is one of the most common endocrinopathies affecting 5-7% of reproductive age women worldwide. The aim of our study was to explore the PCOS-related single nucleotide polymorphism (SNP) associations between common genetic variants and PCOS risk in a Han Chinese women population.

**Methods:**

In this case-control study, 285 Chinese Han women aged 28.50±6.858 years with PCOS and 299 controls of a mean age of 32.66±7.018 years were compared. We selected recently published genome-wide association studies (GWAS) which identified several genetic loci in PCOS. All the SNPs were genotyped by Sequenom Mass-ARRAY technology. Associations between the gene and the risk of PCOS were tested using various genetic models by Statistical Package for the Social Sciences and Plink.

**Results:**

We found that rs705702 in the RAB5B/SUOX was associated with PCOS (odds ratio=1.42; 95% confidence interval=1.08-1.87, p=0.011) and increased the PCOS risk. The genotypic model analysis also showed that rs705702 was associated with PCOS risk.

**Conclusion:**

Our results suggest that SNPs rs705702 in gene RAB5B/SUOX was associated with PCOS in Han Chinese women.

**WHAT IS ALREADY KNOWN ON THIS TOPIC?**

The first genome-wide association studies and subsequent follow-up performed on Han Chinese populations identified the following Polycystic ovary syndrome (PCOS) candidate loci: DENND1A, INSR, YAP1, C9orf3, RAB5B, HMGA2, TOX3, SUMO1P1/ZNF217, THADA, follicle-stimulating hormone receptor, luteinizing hormone/choriogonadotropin receptor.

**WHAT THIS STUDY ADDS?**

Single nucleotide polymorphisms rs705702 in gene RAB5B/SUOX was associated with PCOS in Han Chinese women.

## INTRODUCTION

Polycystic ovary syndrome (PCOS) is the most common reproductive disorder in women. It is a complex, heterogeneous disorder characterized by chronic anovulation, clinical and/or biochemical hyperandrogenism and polycystic ovaries. It is the most common endocrine disorder in premenopausal women and affects up to 10% of them (1). The pathogenesis of PCOS is not fully understood, but it is accepted as a multifactorial disorder that arises from interactions between genetic, environmental, and intrauterine factors (2,3). Several family and twin studies indicate a strong genetic basis and twin studies of heredity provide the most rigorous demonstration that the disorder has a genetic component. Twin studies have provided heritability estimates for PCOS of 70% (4). Several groups have undertaken genetic studies to identify the etiology of PCOS. Genome-wide association studies (GWAS) offer a potentially powerful approach to identify the associations between millions of single-nucleotide polymorphisms (SNPs) and specific traits or disorders (5). The first GWAS and subsequent follow-up performed on Han Chinese populations identified the following PCOS candidate loci: DENND1A, INSR, YAP1, C9orf3, RAB5B, HMGA2, TOX3, SUMO1P1/ZNF217, THADA, follicle-stimulating hormone receptor (FSHR), luteinizing hormone/choriogonadotropin receptor (LHCGR) (6,7). At 19p13.3, rs2059807 is located in the intron of the INSR (insulin receptor) gene and in previous studies, common SNPs in the INSR gene have been reported to be associated with PCOS in both Han Chinese individuals and those of European ancestry. INSR has an important role in insulin metabolism, consistent with a very common explanation for the pathogenesis of PCOS, namely, insulin resistance (8). Mutations affecting the tyrosine kinase domain of the insulin receptor are known to cause severe hyperinsulinemia and insulin resistance (9). LHCGR is expressed on the surface of target cells of reproductive organs such as the ovary, uterus, fallopian tube, and in a variety of other tissues including vascular endothelium (10,11). Studies conducted over the past decade have built a convincing argument that genetic factors contribute to development of PCOS. Despite advances in genetic technologies, very few PCOS susceptibility genes have been validated. To date, there are only a few published genome-wide associated variants for PCOS in Han Chinese women. Indeed, these studies will be important because the susceptibility variants may differ in individual ethnic groups as do the phenotypic features of PCOS. It has been well established that ethnic background adds to the phenotypic diversities in PCOS patients. Although one group recently replicated two of these loci (12), another did not (13). We examined the same susceptibility variants in additional PCOS case-control sets in Han Chinese women. We also investigated variants that correlate strongly with the risk variants in the Chinese population for association with PCOS in the case-control study.

## METHODS

In this case-control study, 584 women were recruited from the Second Affiliated Hospital, Xi’an Jiatong University, during 2009 to 2013. Among these subjects who were all Han Chinese women, 285 were diagnosed as PCOS according to the National Institutes of Health criteria (14). The mean age of this study group was 28.50±6.858 years. None of the subjects had any other diseases and none of them had undergone chemotherapy or radiotherapy. All were unrelated individuals of reproductive age who had received no hormonal therapy for at least three months prior to the study. The control group consisted of 299 healthy women of a mean age of 32.66±7.018 years.

The study was approved by the ethics committee of the Second Affiliated Hospital, Xi’an Jiatong University, and written informed consent was obtained from all participants.

Weight and height were measured in all subjects by standard protocol and calibrated instruments. Body mass index (BMI) was calculated as weight (kg) divided by height squared (m2). Demographic and personal data were collected through a face to face interview using a standardized epidemiological questionnaire, including age, ethnicity, residential region, and family history of PCOS. In addition, relevant information for PCOS patients was collected through consultation with treating physicians or through a review of the medical chart.

A peripheral blood sample of 5 mL was taken from all participants.

### Single-Nucleotide Polymorphism Selection and Genotyping

From the previously published work, we selected 10 SNPs which could be associated with PCOS. Additionally, minor allele frequency (MAF) of these SNPs in the HapMap CHB (Chinese Han Beijing) population was >5%. We used the GoldMag-Mini Whole Blood Genomic DNA Purification Kit (GoldMag Co. Ltd. Xi’an City, China) for the extraction of genomic DNA from whole blood, and DNA concentration was measured by NanoDrop 2000 (Gene Company Limited). We used Sequenom MassARRAY Assay Design 3.0 Software to design Multiplexed SNP MassEXTEND assay (15). We performed Sequenom MassARRAY RS1000 to genotype the SNPs using the standard protocol recommended by the manufacturer (15). Finally, data management and analysis were performed by Sequenom Typer 4.0 Software (15,16). Laboratory personnel were blinded to the genotyping results of all samples.

### Statistical Analysis

Microsoft Excel and Statistical Package for the Social Sciences 19.0 statistical package (SPSS, Chicago, IL, USA) were used to perform the statistical analyses. A p-value <0.05 was considered statistically significant. The validation of each SNP frequency in control subjects was tested for departure from Hardy-Weinberg Equilibrium (HWE) using an exact test. The χ^2^ test was used to compare the distribution of genotypes and allele frequencies between patients and control subjects. The most common genotype in the controls was used as reference group. The associations between the genes and the risk of PCOS were tested using genetic models (co-dominant, dominant, recessive, over-dominant, and log-additive) analysis by SNP stats, website software from http://bioinfo.iconcologia.net/snpstats/start.htm. Odds ratios (ORs) and 95% confidence intervals (CIs) were calculated by unconditional logistic regression analysis adjusted for age and gender (17). We used the Haploview software package (version 4.2) and SHEsis software platform (http://www.nhgg.org/analysis/) for analyses of linkage disequilibrium (LD), haplotype construction, and genetic association at polymorphism loci, and D’ >0.8 indicated that the related tSNPs formed one block (18,19).

## RESULTS

The characteristics of PCOS and control subjects are presented in Table 1. The PCOS group was older than the control group (p<0.001). There were no significant differences in BMI (p=0.585). As listed in Table 2, a multiplexed SNP MassEXTEND assay was designed with the Sequenom MassARRAY Assay Design 3.0 Software. No significant deviation of allele frequencies from HWE was found in PCOS and control groups. We investigated the possibility of the minor allele of each SNP being a risk factor compared with the wild-type allele. As shown in Table 3, the allele and genotype frequencies of the ten SNP were not significantly different between PCOS patients and healthy controls. As shown in Table 3, we found that rs705702 in the SUOX was associated with PCOS (OR=1.42; 95% CI=1.08-1.87, p=0.011) and increased the PCOS risk. The genotypic model analysis showed that rs705702 was associated with PCOS risk (Table 4) and logistic regression analysis adjusted for age and BMI also showed an association with PCOS risk. Using the AA genotypes combined as a reference, the AG/GG genotype of rs705702 was significantly associated with an increased risk of PCOS (adjusted p=0.018, OR=1.52; 95% CI=1.07-2.16, Dominant model). In the log additive model, we found that rs705702 was significantly associated with an increased risk of PCOS (adjusted p=0.014, OR=1.42; 95% CI=1.07-1.98). Since the pattern of LD is highly structured into conserved blocks of sequence separated by hotspots of recombination, the final function of a conserved haplotype may be the result of interaction among polymorphisms within the block. We analyzed linkage LDs among the SNPs and these SNPs showed no tight links.

## DISCUSSION

In this case-control study, we aimed to determine whether variants recently identified in a GWAS for PCOS in Chinese Han subjects would be associated with PCOS in Xi’an Chinese Han people. This is the first study to report the frequencies of genotypes and alleles in ten different SNPs (rs13405728, rs3802457, rs1894116, rs705702, rs2272046, rs1961177, rs1048943, rs4784165, rs2059807, rs6022786), which were identified by GWAS, between PCOS and healthy Xi’an Han ethnic people. Our results also showed that rs705702 in the RAB5B/SUOX was associated with PCOS risk, while other loci were not found to be significantly associated with PCOS risk. The different genotype distributions might reflect differences in genetic background, and therefore gene variants might be associated with different relative risks in different populations. Although a previous study demonstrated that these SNPs were associated with PCOS in Han Chinese women, the current study showed that these SNPs were not involved in the pathogenesis in Xi’an Han Chinese women. These different results indicated that there is different genetic background between these SNPs and PCOS.

rs705702 is located at 12q13.2 between the RAB5B and SUOX genes. RAB5B, PCOS GWAS candidate, is a Rab-GTPase, also thought to be involved in endocytosis and receptor recycling and could, therefore, be a molecule interacting with the DENN domain (20,21,22) and has also been reported to involve PI3K, PKB, and MAPK/ERK components (23). RAB5B is a small GTPase that plays a role in early endosome formation and is required for the endocytic pathway that mediates the transport of clathrin-coated vesicles from the plasma membrane to the early endosome. RAB5B is a member of the RAS oncogene family and SUOX, sulfite oxidase, is a homodimeric protein enzyme localized to the intermembrane space of mitochondria, which catalyzes oxidation of sulfite to sulfate, the final reaction in the oxidative degradation of the sulfur amino acids cysteine and methionine. rs705702 was not associated with PCOS in the current study and was only nominally significant in previous European studies (13). Shi et al (7) found that rs705702 in RAB5B/SUOX was associated with PCOS in the Han Chinese study and in our current study, we also showed that it was associated with PCOS in Xi’an Han Chinese people. As to other loci, in our study, we found no significant correlations with PCOS. However, in the GWAS study, it was reported that rs13405728 (LHCGR), rs3802457 (C9orf3), rs1894116 (YAP1), rs2272046 (HMGA2), rs4784165 (TOX3), rs2059807 (INSR), and rs6022786 (SUMO1P1) were associated with PCOS risk in Han China (7). In addition, rs13405728 was shown to have an association with PCOS in Hui Chinese people (12). rs3802457 was shown to be related to PCOS in a Dutch study (4). As to other loci, an association with PCOS risk has not been reported in studies from Europe. Therefore, the ultimate role of this variant in PCOS pathogenesis remains to be determined.

To conclude, our study has, for the first time, described an association between SNPs in RAB5B and PCOS risk in a group composed of Han individuals of Xi’an China. Large well-designed and population-based studies are warranted to confirm these findings which may well have implications for the etiology of PCOS.

## Acknowledgments

This work is supported by the Shaanxi Provincial scientific and technological research projects S2015YFSF0310. We are grateful to all the patients and individuals in the study who made this work possible. We would also like to thank the clinicians and hospital staff who contributed to data collection for this study.

## Ethics

Ethics Committee Approval: Second Affiliated Hospital, Xi’an Jiatong University during 2009 to 2013, Informed Consent: Written informed consent was obtained from all participants.

Peer-review: Externally peer-reviewed.

## Figures and Tables

**Table 1:**
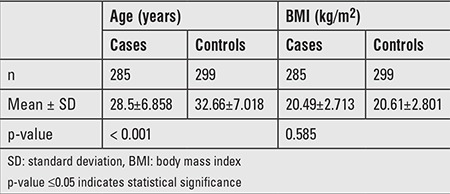
Clinical characteristics of the Polycystic ovary syndrome cases and of the control group

**Table 2:**
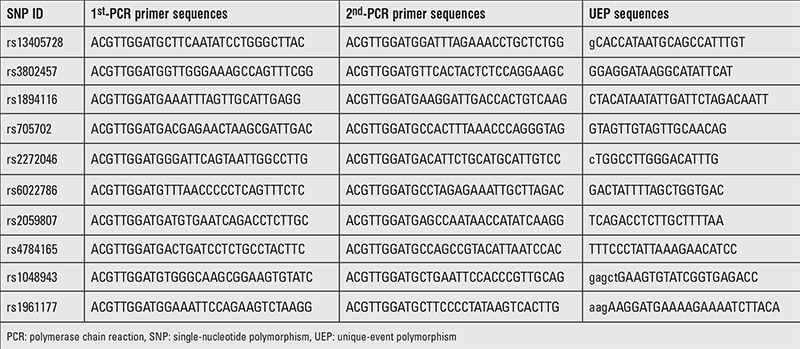
Polymerase chain reaction primers

**Table 3:**
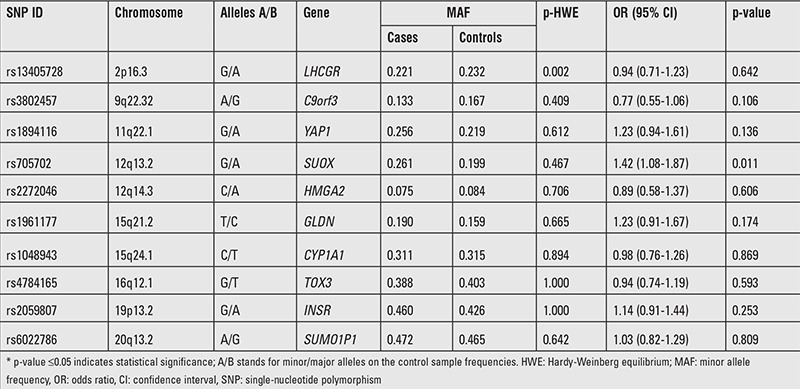
Basic information of candidate single-nucleotide polymorphisms in this study

**Table 4:**
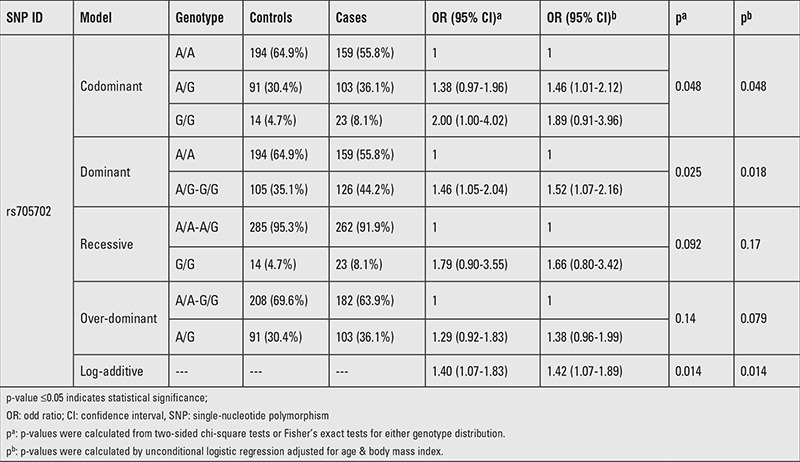
Association between rs705702 and Polycystic ovary syndrome risk
